# Supplementary material for: MUTYH Activity Maintains Telomere Stability in Response to Chronic Telomeric 8-Oxoguanine Damage in Cancer Cells
Source: Biomolecules. 2026 Aug 26;16(9):1234. doi: 10.3390/biom16091234 (PMC13604166; doi:10.3390/biom16091234)
Supplement: Supplementary file 1 [file biomolecules-16-01234-s001.zip › biomolecules_4443357_supplementary figures.pdf]

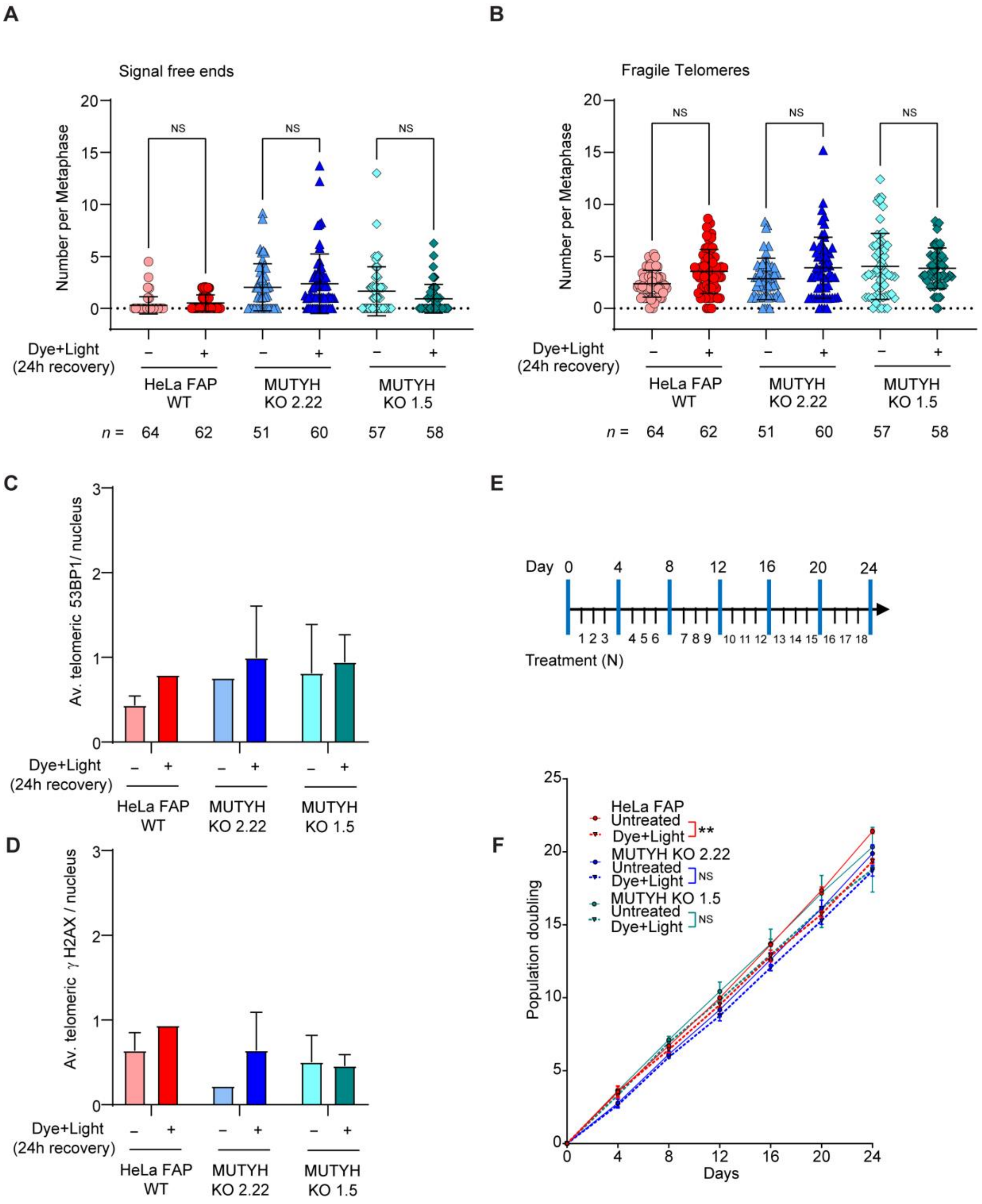


**Figure S1. MUTYH deficiency does not increase telomere instability after acute telomeric 8-oxoG damage or impair cell growth after chronic damage. (A–B)** Number of telomeric signal-free chromatid ends (**A**) or fragile telomeres (B) per metaphase in the indicated cell lines, untreated or treated with 5 min dye + light and recovered 24 h (acute exposure to telomeric 8-oxoG). Data represent the mean ± s.d. from the indicated n number of metaphases analyzed from three independent experiments, normalized to the chromosome number. One-way ANOVA; ns = non- significant. **(C–D)** Quantification of average telomeric 53BP1 (**C**) and telomeric γH2AX (**D**) per nucleus 24 h after treatment with 5 min dye + light. Data represent the Mean ± SEM from two independent experiments; one-way ANOVA, ns = non-significant. **(E)** Schematic of the repeated telomeric 8-oxoG inductions. Blue bars indicate days when cells were harvested and not exposed to 5 min of dye + light. **(F)** Population doubling (PD) over 24 days of untreated cells (solid line) and cells treated with dye + light each day except every 4th day of harvest (dotted line). Data represent the mean ± SEM from three independent experiments. Welch's two-sample t test comparing untreated and dye + light at N18 per each cell line; ** $p < 0.05$.

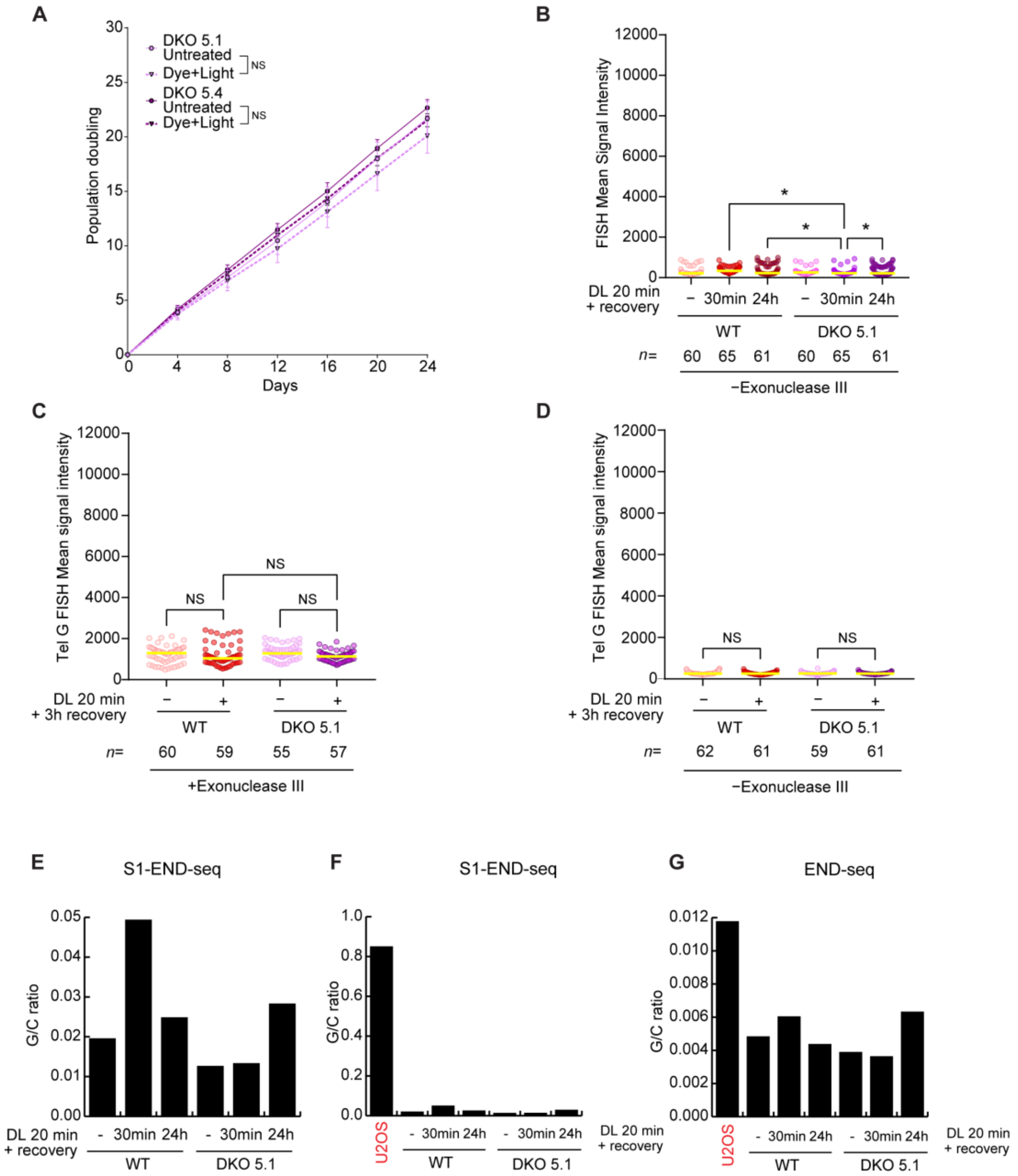


**Figure S2. Growth of doubly glycosylase deficient cells after chronic telomere damage and controls for SSB detection experiments.** (**A**) PD over 24 days of untreated cells (solid line) and cells treated with 5 min dye + light each day except every 4th day of harvest (dotted line). Data represent the mean ± SEM from three independent experiments. Welch's two-sample t test comparing untreated and dye + light at N18 per each cell line; ns = non-significant. (**B**) Quantification of exo-FISH signal intensity control from Figure 5A-B without exonuclease III treatment. Each data point represents the mean fluorescence intensity for each nucleus. Data represent the median of the indicated *n* number of nuclei analyzed from three independent experiments; * $p < 0.05$, one-way ANOVA. **(C–D)** Quantification of exo-FISH signal intensity from cells untreated or treated with 20 min dye + light and recovered for 3 h, with (**C**) or without (**D**) exonuclease III treatment. Each data point represents the mean fluorescence intensity for each nucleus. Data represent the median of the indicated *n* number of nuclei analyzed from three independent experiments; ns = non-significant, one-way ANOVA. **(E)** Ratio of G-rich to C-rich telomeric reads (G/C), not normalized on the untreated samples, obtained by S1-END-seq in untreated and dye + light treated cells recovered for the indicated times. An increase in the G/C ratio reflects enrichment of S1-sensitive single-stranded regions within telomeres, indicative of accumulated repair intermediates. **(F-G)** Ratio of G-rich to C-rich telomeric reads (G/C), not normalized on the untreated samples, obtained by S1-END-seq (**F**) and END-seq (**G**) in untreated and dye + light treated U2OS cells recovered for the indicated times. U2OS cells were used as a control cell line for comparison since they harbor elevated telomeric SSBs and DSBs.

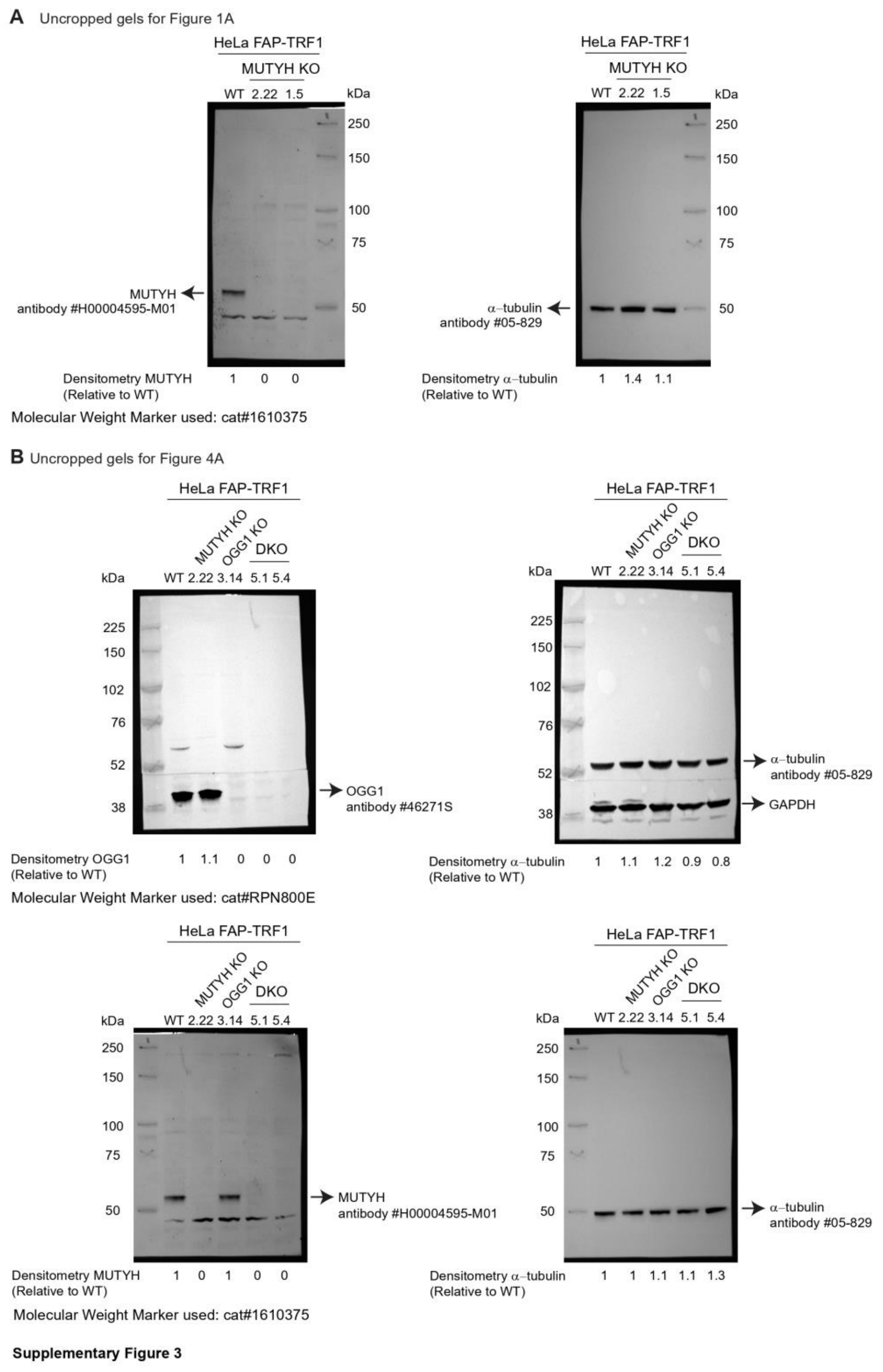


**Figure S3. Images of uncropped gels.**
